# A Component of Retinal Light Adaptation Mediated by the Thyroid Hormone Cascade

**DOI:** 10.1371/journal.pone.0026334

**Published:** 2011-10-24

**Authors:** Diana E. Bedolla, Vincent Torre

**Affiliations:** 1 Neurobiology Sector, International School for Advanced Studies (SISSA), Trieste, Italy; 2 Italian Institute of Technology (IIT), SISSA-Unit, Trieste, Italy; Clermont Université, France

## Abstract

Analysis with DNA-microrrays and real time PCR show that several genes involved in the thyroid hormone cascade, such as deiodinase 2 and 3 (*Dio2* and *Dio3*) are differentially regulated by the circadian clock and by changes of the ambient light. The expression level of *Dio2* in adult rats (2–3 months of age) kept continuously in darkness is modulated by the circadian clock and is up-regulated by 2 fold at midday. When the diurnal ambient light was on, the expression level of *Dio2* increased by 4–8 fold and a consequent increase of the related protein was detected around the nuclei of retinal photoreceptors and of neurons in inner and outer nuclear layers. The expression level of *Dio3* had a different temporal pattern and was down-regulated by diurnal light. Our results suggest that DIO2 and DIO3 have a role not only in the developing retina but also in the adult retina and are powerfully regulated by light. As the thyroid hormone is a ligand-inducible transcription factor controlling the expression of several target genes, the transcriptional activation of *Dio2* could be a novel genomic component of light adaptation.

## Introduction

Photoreceptor cells and retinal neurons tune their properties according to the ambient illumination and the circadian rhythm [1], [2]. Indeed, several mechanisms are affected by the circadian clock and the intensity of the ambient light, such as the rate of disk shedding [3], the expression level of genes such as *c-fos*, *c-jun*, *jun B*, transducin and rhodopsin [4], [5] and several other biochemical and physiological properties [6]. All these mechanisms allow retinal neurons to optimally adapt to the circadian clock and to prolonged changes of ambient light, not associated to those naturally occurring during the circadian clock.

Photoreceptors and retinal neurons are able to operate over a wide range of light intensities, approximately 10 log units, because of light adaptation. In photoreceptors, light adaptation has been extensively studied and several mechanisms contribute to it: changes of intracellular calcium concentration [6]–[11]; the light-driven redistribution of transducin and arrestin between the outer and the inner segment [12]–[15] leading to a reduction in photoreceptor sensitivity and thus to light adaptation. Recently, it has been shown that changes in gene expression in photoreceptors could also contribute to light adaptation: a consistent up-regulation of almost two-fold of arrestin (*Sag*) [16], guanylyl cyclase activating protein 1A (*Guca1a* also known as *Gcap1*) [17], [18] and guanylyl cyclase activating protein 1B (*Guca1b* also known as *Gcap2*) [17], [18] has been observed in isolated rods and intact retinas [19].

In the present manuscript, we analyze changes in gene expression occurring during the circadian rhythm and when the ambient light in the circadian rhythm is modified. A microarray analysis identified the gene coding for the DIO2 enzyme as the gene with the largest changes of expression levels. This observation prompted us to investigate whether the observed DNA microarray results could be confirmed with real time PCR and if the corresponding protein levels are also modified after light exposure. Several reports have described a major role of the thyroid hormone cascade during retinal development [20]–[23] and recently also in adulthood [24]. The active form of the thyroid hormone, triiodothyronine – usually referred to as T3 – binds the thyroid hormone receptor and activates it. The level of T3 is increased by deiodination of thyroxine (T4) catalyzed by type 2 deiodinase (DIO2) and is decreased by a further deiodination of T3 catalyzed by type 3 deiodinase (DIO3) [25]–[28]. T3 mediates the activation of nuclear thyroid hormone receptors, TRα and TRβ, ligand-inducible transcription factors regulating a variety of target genes [29]. Given the protective action of the thyroid hormone cascade on the survival and maturation of cone photoreceptors [30], we asked whether activation of the thyroid hormone cascade could be a component of light adaptation.

## Results

We measured changes in gene expression in retinas acutely isolated from adult (3 months old) freely moving rats exposed to controlled ambient steady lights. To determine the amount of light impinging on the retina, we performed a similar analysis also in cultivated intact retinas [31] – where the flux of photons was precisely measured – but these retinas exhibited a progressive loss of integrity over 24 hours. Therefore, we decided to use freely moving rats to establish the effect of the circadian clock and prolonged light exposures on gene expression levels in the retina.

### Microarray analysis of changes in gene expression

**Figure 1:**
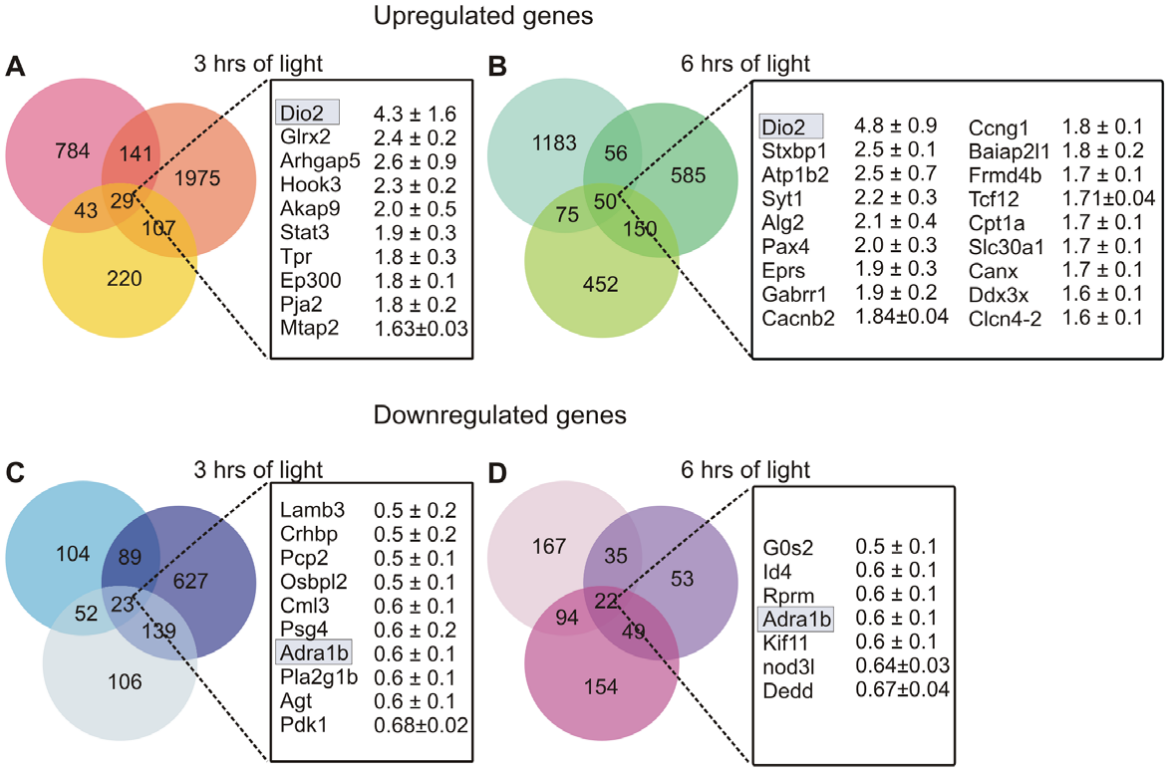
Genes that showed an up- or down-regulation. Genes up-regulated of at least 1.6 fold at (A) 3 hours and (B) 6 hours of light exposure. The genes that showed an up-regulation in the three replicas are listed and the only gene that appears in both lists is *Dio2*. Genes that showed a down-regulation less than 0.7 at (C) 3 hours and (D) 6 hours of exposure to light. The only gene that showed a down-regulation at both timings was *Adra1b*. Each circle represents one replica. The numbers in each set represent genes that are identified, predicted genes or transcribed locus. In the list, only the identified ones are mentioned.

We performed an initial screening using the DNA microarray technique. We extracted the mRNA from retinas of rats kept overnight in darkness until 7 am (as control) 0 ZT and subsequently retinas of animals that were exposed either for 3 hours (3 ZT) or 6 hours (6 ZT) to a 1000 lux light. From the 31099 probes present in the microarray, we extracted those whose expression level increased by more than 60% in all three replicas (Fig. 1). Up-regulated genes after 3 (3 ZT) and 6 hours (6 ZT) of light exposure were 29 and 50 respectively. *Dio2* was the maximally up-regulated gene after 3 and 6 hours of light and its up-regulation was consistently observed in all replicas. Since DIO2 regulates the availability of the active thyroid hormone, as a consequence, DIO2 regulates the timing of cellular responses to thyroid hormones [32].

Three transcription factors were up-regulated both after 3 and 6 hours of light: *Stat3* (signal transducer activator of transcription 3), *Ep300* (E1A binding protein p300) and *Pax4* (paired box gene 4) involved in retinal transcription [33]–[35]. *Stat3* and *Ep300* are involved in the thyroid hormone cascade as downstream transcription factors [36].

We performed a gene ontology analysis of up-regulated genes (http://bioinfo.vanderbilt.edu/webgestalt/). Among up-regulated genes we found 11 genes involved in visual functions and eye development: *Psen1* (presenilin 1), *Crygb* (crystallin, gamma B), *Crygc* (crystallin, gamma C), *Crygd* (crystallin, gamma D), *Grk1* (G protein-coupled receptor kinase 1), *Rpgrip1* (retinitis pigmentosa GTPase regulator interacting protein 1), *Myo5a* (myosin Va), *Stat3* (signal transducer and activator of transcription 3), *Pax4* (paired box 4). These genes could be involved in the protection of the retina during exposure to bright lights.

There were 18 up-regulated genes involved in cell-to-cell communication, synaptic function and transmission of nerve impulse. Among them we found *Gabrr1* (gamma-aminobutyric acid (GABA) receptor, rho 1) and *Cacnb2* (calcium channel, voltage-dependent, beta 2 subunit). *Gabbr1* codes for the GABA receptor subunit rho1, one of the subunits particular of GABA_C_ receptors highly expressed in the retina [37]–[39]. *Cacnb2* corresponds to the beta subunit of a voltage gated calcium channel known to modulate the b-wave of the ERG response in dark [40]

Up-regulated genes involved in general cell functions and metabolism were *Atp1a1* (ATPase, Na+/K+ transporting, alpha 1 polypeptide), *Scarb1* (scavenger receptor class B, member 1), *Crh* (corticotropin releasing hormone) and *Dio2* (deiodinase, iodothyronine, type II).

Down-regulated genes were those that had a decreased expression level larger than 0.7 and were 23 and 22 after 3 and 6 hours of light exposure for all three replicas (Fig. 1C and 1D), respectively. The only gene down-regulated at both times was *Adra1b* (adrenergic receptor, alpha 1b), coding for the alpha 1b adrenergic receptor involved in the control of cyclic AMP (cAMP) when epinephrine is present [41]. cAMP regulates proteins involved in cone photoresponses via G protein-coupled receptor kinases [42]. Therefore, *Adra1b* could have an important role in light adaptation.

### Light and circadian regulation of genes Dio2 and Dio3

The gene which was consistently and prominently up-regulated after 3 and 6 hours of light was *Dio2*. As a consequence, we decided to confirm changes in the expression of genes involved in the production or reduction of thyroid hormone *Dio2* and *Dio3* with real-time PCR in retinas extracted from freely moving rats kept in different ambient lights.

**Figure 2:**
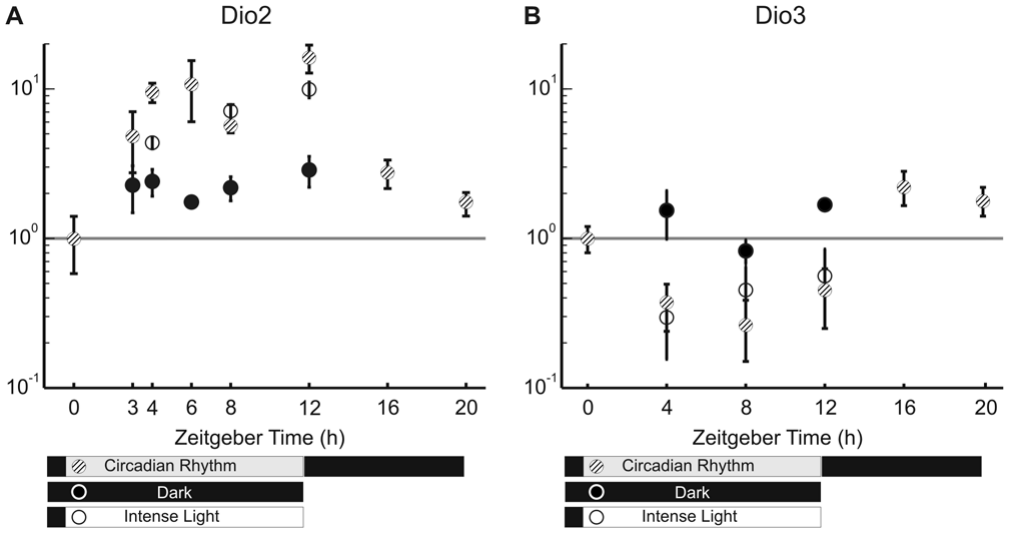
Expression of *Dio2* and *Dio3* using real time PCR. (A) Expression of *Dio2* showing an up-regulation due to the time of the day (compared to Dark) and as response to light (Circadian Rhythm and Intense Light). (B) Expression of *Dio3* showing a down regulation mainly due to light. Data are reported as mean ± S.E.M (N = 6).

Rats were kept in darkness from 7 pm to 7 am and in ambient light conditions equivalent to 600 Lux from 7 am to 7 pm. This setting is here referred to as the circadian rhythm (indicated by striped dots in Fig. 2). In some experiments, rats were kept in complete darkness for an entire day (indicated by dark dots in Fig. 2) and their retinas were extracted at specific times. In other experiments, rats were exposed to a more intense ambient light equivalent to 1000 Lux from 7 am to 7 pm (or the specified time indicated by white dots in Fig. 2). Changes in gene expression at each time were obtained by pooling at least 6 rat retinas. The reference level of gene expression was taken as that measured at 7 am, i.e. at ZT 0 (Zeitgeber Time). In rats kept in darkness after 7 am and therefore not exposed to the usual diurnal light, the expression level of *Dio2* increased by about 2 times at ZT 3, ZT 4, ZT 6, ZT 8 and ZT 12. The expression level of *Dio2* in rats exposed to the usual light or to an intense light, from ZT 0 increased with time of light exposure, reaching levels 4–8 times larger than in control conditions (Fig. 2A). We could not detect any significant difference when rats were kept in cages illuminated with the usual ambient light or a more intense light. During the usual night darkness, the level of *Dio2* returned progressively to the original level (Fig. 2A).

An opposite pattern was observed for the expression level of *Dio3*: in rats exposed to the usual diurnal light or more intense light, the expression level of *Dio3* decreased by about two times (Fig. 2B). The level of *Dio3* did not change significantly in rats continuously kept in darkness (Fig. 2B) suggesting that the observed decrease of expression is primarily caused by the light and not by the intrinsic circadian rhythm. The relative abundance of Dio2/Dio3 was estimated by using the ΔCt method, normalizing with respect to the housekeeping reference genes. As shown in table S1, the expression ratio between *Dio2* and *Dio3* increases up to 10-fold at 8 ZT, during normal day light illumination and decreased in the dark during the night.

### Protein changes during the circadian regulation

**Figure 3:**
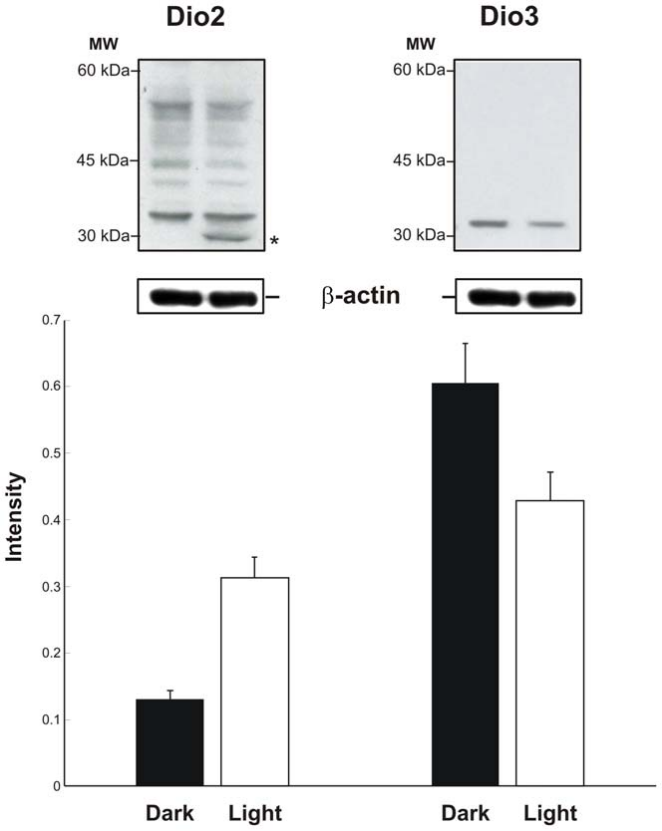
Western blot corresponding to DIO2 and DIO3 showing an agreement with real time PCR results. (A) Representative immunoblot corresponding to DIO2 and DIO3. Although DIO2 shows non-specific bands, a protein band of estimated molecular weight is clearly present in rats exposed to light and absent in dark. MW: Molecular Weight. *:band corresponding to DIO2. (B) Histograms of densitometric measurements of the western blots (N = 3) All band intensities of each protein were compared separately with that of β-actin. The specificity of both antibodies can be observed.

In order to verify whether up-regulation of *Dio2* and down-regulation of *Dio3* genes resulted in an increased (or decreased) level of related protein, the expression levels of associated proteins were determined by Western blot (Fig. 3) from retinas of freely moving adult rats kept in darkness and of rats exposed to a steady bright light equivalent to 1000 lux for 3 hours. Immunoblot revealed bands for both proteins at 30 kDa. Although the DIO2 antibody was not highly specific and recognized several non-specific bands (Figure 3A), a clear protein band of molecular weight of 30 kDa corresponding to DIO2 was present in samples obtained from rats exposed to light and absent in samples from rats kept in darkness. The antibody for DIO3 was more specific and only the band corresponding to the molecular weight of DIO3 was observed. Western blot analysis using densitometric measurements normalized to β-actin showed that the concentration of DIO2 increased by 131% and of DIO3 protein decreased by 30% (Fig. 3). Therefore, observed changes in gene expression were associated to concomitant changes of protein synthesis.

### Retinal localization of the protein deiodinase 2

**Figure 4:**
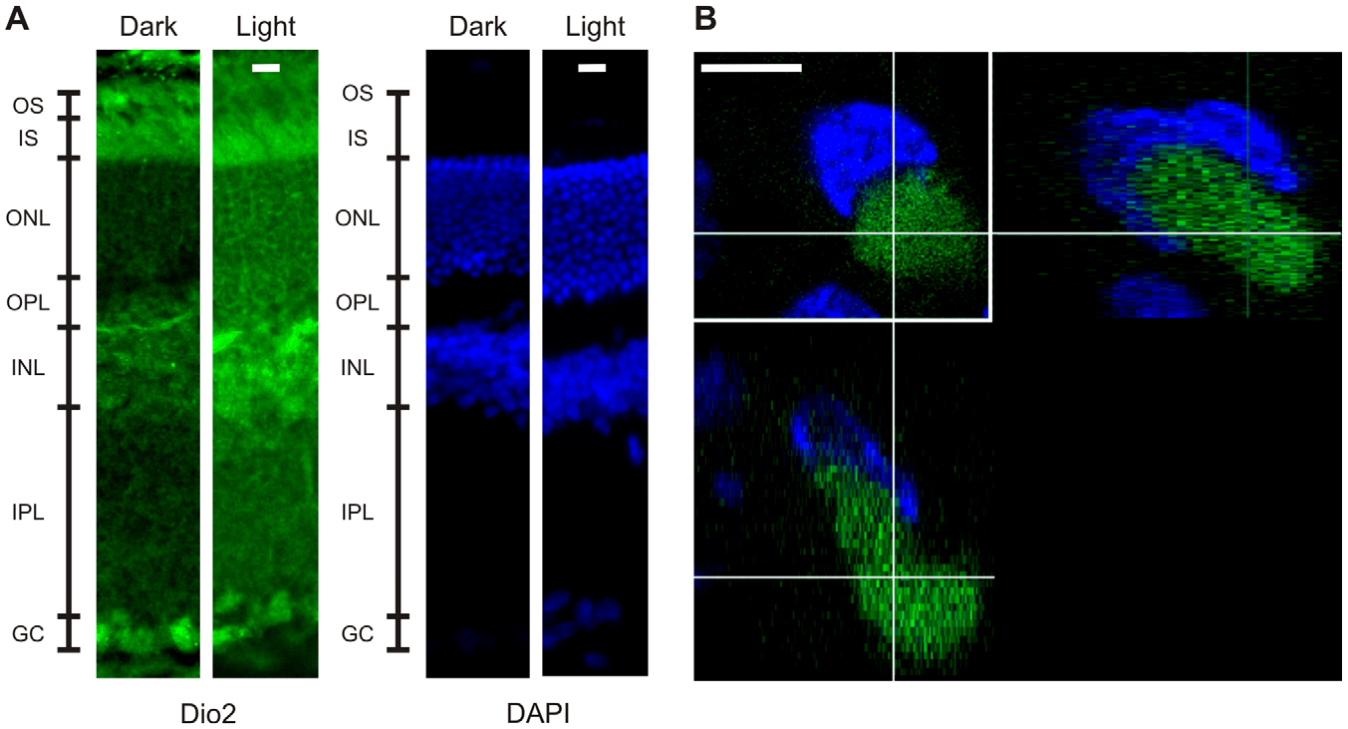
Immunofluorescence of adult rat retina in dark and light conditions (3 hours of steady illumination). (A) In blue for the nuclei (DAPI) and in green for DIO2. (OS photoreceptor outer segment; IS inner segment; ONL outer nuclear layer; OPL outer plexiform layer; INL inner nuclear layer; IPL inner plexiform layer; GC ganglion cells). Scale bar = 10μm. (B) Inner nuclear layer nucleus in blue, do not colocalize with DIO2 in green, in the right side the y–z plane, and in the lower left the x–z plane. Scale bar = 5 µm.

In order to determine the location where the increase of DIO2 occurred in the retina, we used immunofluorescence imaging with a confocal microscope. In darkness (Fig. 4A), staining for DIO2 (in green) was observed at the level of inner (IS) and outer segments (OS) of photoreceptors, in the inner nuclear layer (INL) and in the ganglion cells (GC). In light adapted conditions (3 hours of continuous light), staining for DIO2 was diffused over the whole retina and in particular in photoreceptor inner segments, the outer nuclear and plexiform layer (ONL, OPL) and in the INL. The increased staining for DIO2 was seen around the nuclei, in agreement with the known localization of DIO2 in the endoplasmic reticulum (ER) [28]. DIO2 is expected to increase the level of T3 in the cytosol with ready access to the nucleus due to the physical proximity of the nuclear compartment to the ER [28]. Confocal images taken at a higher magnification (Fig. 4B) show that in the inner plexiform layer, DIO2 (in green) is localized in close proximity to the nuclei (in blue), in agreement with the notion that DIO2 is an ER resident protein generating T3 in the cytosol [28].

### Light regulation of target genes of the thyroid hormone cascade

**Figure 5:**
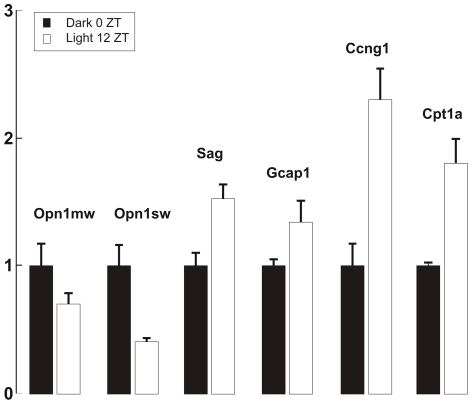
Expression of *Opn1mw*, *Opn1sw*, *Sag*, *Gcap1*, *Ccng1*, *Cpt1a*. Dark columns show the data of rats kept in dark overnight until 7 am (0 ZT). White columns show data after 12 hours of ambient light (12 ZT). Data reported as mean ± S.E.M (N = 3).

The up-regulation of *Dio2* caused by light exposure is expected to increase the concentration of the active thyroid hormone T3 and if the thyroid hormone cascade plays a role in light adaptation, target genes known to be controlled by T3 should also be controlled by light. In human WERI-Rb1 cell line, T3 regulates two genes involved in phototransduction, i.e. *Sag* and *Gcap1* [43] coding for proteins involved in light adaptation. The maximum change in expression of *Dio2* happens between 0ZT and 12 ZT (Fig.2), therefore, we verified by real time PCR the effect of light exposure on these genes using samples obtained at 0 ZT after being kept overnight in the dark and 12 ZT after 12 hours of light exposure. As shown in Fig.5, *Sag* and *Gcap1* were clearly up-regulated at 12 ZT as expected.

Several investigations report that T3 regulates also genes coding for the medium and long wavelength cone opsins (*OPN1LW*/*OPN1MW*) in human WERI-Rb1 cell lines [43]. Glaschke et al. found that in adult rodent retinas, T3 controls the expression of medium and short wavelength cone opsin (*Opn1mw* and *Opn1sw*) [44]. On the other hand, the activation of the thyroid hormone receptor β2 (TRβ2) down-regulates the expression of *Opn1sw* and *Opn1mw* [45]–[47]. In this work, we observe a similar down-regulation in intact rats caused by light and in fact at 12 ZT under light exposure, the expression level of *Opn1sw* and of *Opn1mw* was respectively about 30% and 40% lower than at 0 ZT in the dark (Fig.5).

Thyroid hormone receptors, TRα and TRβ, regulate target gene expression by binding to the T3 response element (TRE) composed of repeated DNA sequences with different configurations. The consensus sequence recognized by nuclear receptors often contains a hexamer AGGTCA known as “the half site”. TR forms heterodimers with members of the retinoid X receptor (RXR) family to mediate T3 action [36]. TR/RXR activates through the DR4 element (two half sites in the same orientation spaced by four base pairs), that is AGGTCANNNNAGGTCA. To find additional target genes of the hormone cascade, we scanned their promoter sequences (downloaded from http://www.mybioinfo.info/) to locate the existence of a DR4 element. We found that *Atp1b2* (ATPase, Na^+^/K^+^ transporting, beta 2) contains this exact sequence and *Ep300* (E1a binding protein), *Ccng1* (cyclin g1) and *Cpt1a* (carnitine palmitoyltransferase 1a, liver) contained a sequence that had the two half sites but spaced by 5, 6, 6 base pairs, respectively. TR binding and weak transactivation to sequences with 5 and 6 base pairs between the two half sites has been reported [27]. *Cpt1a* is known to be regulated by T3 [48], and regulates some cyclins [49]. We were unable to verify the expression of *Atp1b2* and *Ep300*, but the two genes *Ccng1* and *Cpt1a*, were up-regulated also by light, as shown in Fig.5. *Ccng1* could have a protective role and in cellular survival as reported for other cyclins [50], [51], and *Cpt1a* play a role in retina metabolism [52].

These results support the notion that light could activate the thyroid hormone cascade, regulating therefore the expression level of its target genes, such as the cone opsins, *Sag* and *Gcap1*. These biochemical pathways could be novel components of light adaptation.

## Discussion

Our results demonstrate that the circadian clock and the ambient light influence the expression level of *Dio2* and *Dio3* genes and of their corresponding proteins in the adult retina. The genomic analysis of changes in gene expression with DNA-microarrays in the adult retina shows that *Dio2* is the most up-regulated gene by diurnal light (Fig. 1). These results suggest a role of the thyroid hormone cascade during light adaptation in the adult retina, not previously considered.

### The thyroid hormone cascade

The thyroid gland secretes the poorly active compound thyroxine (T4). The relative concentrations of T4 and T3 and their availability to the nuclear thyroid hormone receptor (TR) are controlled by the local conversion of T4 to T3 catalyzed by the enzyme DIO2, while the enzyme DIO3 inactivates T3 [26], [53]. T3 mediates the activation of nuclear thyroid hormone receptors, TRα and TRβ, ligand-inducible transcription factors regulating a variety of target genes [29]. Therefore, the transcriptional activation of *Dio2* is expected to activate the thyroid hormone cascade and thus, to modulate the associated target genes.

In the pituitary gland, *Dio2* and *Dio3* exhibit a regulation of gene expression similar to the one described here in the retina. Several genes present in the retina and in the pineal gland show a phase shift with respect to each other [54], similar to what observed for *Dio2* and *Dio3*. *Dio2* has a role in photoperiodic modulation in seasonal reproduction in the mediobasal hypothalamus [55], [56].

The thyroid hormone cascade acts in the regulation of neurodevelopment, possibly by activation and repression of complex gene networks [57] and DIO2 plays a crucial role during retinal development [20]–[23]. The thyroid hormone stimulates the up-regulation of red-green, violet opsins and rhodopsin and calbindin in photoreceptors in development [58]. Photoreceptors mature expressing photopigments when the thyroid hormone increases in embryonic development [59]. DIO3 acts as a limiting factor to the hormonal exposure of cones to levels that safeguard cone survival and patterning of opsins required for cone function [30].

TR regulates target gene expression by binding to the T3 response element (TRE) composed of repeated DNA sequences with different configurations. The consensus sequence recognized by nuclear receptors often contains a hexamer AGGTCA known as “the half site”. TR forms heterodimers with members of the retinoid X receptor (RXR) family to mediate T3 action [36]. TR/RXR activates through the DR4 element (two half sites in the same orientation spaced by four base pairs), that is AGGTCANNNNAGGTCA. To verify whether up-regulated genes (Fig. 1A–B) could be directly regulated by TR, we scanned their promoter sequences (downloaded from http://www.mybioinfo.info/) to locate the existence of a DR4 element. We found that *Atp1b2* (ATPase, Na+/K+ transporting, beta 2) contains this exact sequence and *Ep300* (E1a binding protein), *Ccng1* (cyclin g1) and *Cpt1a* (carnitine palmitoyltransferase 1a, liver) contained a sequence that had the two half sites but spaced by 5, 6, 6 base pairs, respectively. TR binding and weak transactivation to sequences with 5 and 6 base pairs between the two half sites has been reported [27]. It has been implied that *Ep300* has a role in TR function [60]. The fact that this gene, out of the four we mentioned, has a role in TR function strongly supports the idea that the other three genes depend on the activity of the thyroid hormone cascade and that could also be involved in light adaptation.

### Knock-out mice of Dio2 and Dio3 and Graves' disease

*Dio2* ^−/−^ knock-out mice had significant deficits in thermoregulation and thermogenesis [53], [61]–[63], in skeleton, brain and in auditory functions [64]–[66]. These mice had an almost normal level of T3 and their general health appeared to be good [67]. *Dio*1^−/−^ and *Dio2* ^−/−^ knock-out mice had significant deficits in the Morris water maze test indicating dysfunctions not only in learning and memory development but also, possibly, in visual capability [67]. In *Dio*3^−/−^ knock-out mice, almost 80% of cones are lost through neonatal cell death and the amplitude of both the a- and b-wave of the electroretinogram is significantly reduced [30]. These results suggest that the thyroid hormone cascade contributes to the regulation of retinal functions. However, the almost normal level of T3 in these mice suggests the existence of compensatory mechanisms, likely to mask the exact role of *Dio2* and *Dio3* in retinal visual functions.

In humans, the majority of patients with dysthyroid eye disease (Graves' disease), an autoimmune disease where the thyroid is overactive, producing an excessive amount of thyroid hormones have developed color vision defects [68], in agreement with a possible influence of the thyroid hormone cascade on color vision.

### Comparison with previous investigations

Liu et al [43] analyzed changes in gene expression, in human retinoblastoma cell line (WERI-Rb1) induced by a high level of T3. WERI cells are an early stage cone lineage cell line [69] and these cells express L- and M- opsin in a mutually exclusive pattern, similar to the human retina [70], therefore, this investigation provides a good model of the role of the thyroid hormone cascade in the retina. Changes in gene expression in WERI cells exposed to a high level of T3 were analyzed with DNA microarray and real time PCR, providing a screen of genes modulated by T3. The genes most up-regulated were *OPN1LW* and *OPN1MW*, i.e. the long (L) and medium (M) cone opsin genes and were identified as transcriptional targets of the thyroid hormone cascade. Also *ARR3* (arrestin 3, retinal), *GCAP1, PDE6H* (phosphodiesterase 6H) and *PDE6C* (phosphodiesterase 6C) were found to be similar transcriptional targets. Arrestin, guanylyl cyclase, and phosphodiesterase are proteins involved in the regulation of the cyclic GMP signal transduction pathways in cones and rods [71] and it is remarkable that they are all transcriptional targets of the thyroid hormone cascade. Also *Crx,* the cone-rod homeobox [43] was found to be another transcriptional target of T3 and *OPN1LW*, *Opn1mw*, *Arr3* and *Gcap1* have been identified to be regulated by *Crx* [34], [43], [72], [73]. Recently, Glaschke et al. have shown that thyroid hormone controls cone opsin expression and that *Dio2* and *Dio3* have a similar behavior in wild-type mice treated with MMI/perchlorate, treatment causing mice to become hypothyroidic [24]. In agreement with this observation, genes coding for the cone opsins *Opn1mw* and *Opn1sw* are down-regulated after 12 hours of light exposure (Fig. 5).

In a previous investigation [19], we have shown that exposure to bright light caused an up-regulation of three genes involved in phototransduction in retinal rods. Indeed, during light adaptation, we have observed an up-regulation of almost two-fold of *Sag*, *Gcap1* and *Gcap2* [19]. As shown in Fig. 4A, we observed an increase of the protein DIO2, usually associated to an elevation of T3, in photoreceptor inner segments. These observations suggest the possibility that the thyroid hormone cascade could be involved in the changes of *Sag*, *Gcap1* and *Gcap2* expression observed in retinal rods during prolonged light exposures as shown in Fig. 5 after 12 hours of light exposure.

### Possible role of the thyroid hormone cascade during light adaptation

The present and previous investigations [19], [43] indicate a possible role of the thyroid hormone cascade during light adaptation in the retina. As shown in Fig. 4, prolonged light exposures increase the level of DIO2 throughout the retina and in the soma of retinal photoreceptors. An increased level of DIO2 is expected to enhance the local concentration of T3 activating the thyroid hormone cascade modulating its target genes such as *OPN1LW*, *Opn1mw*, *Opn1sw*, *Arr3* and *Gcap1* [61] controlling light adaptation in photoreceptors. Among genes up-regulated by light (Fig. 1), there is *Pax4*, a homeobox gene, usually involved in developmental events and in adult tissues undergoing frequent renewal [74]. *Pax4* has been found to be expressed in the adult retina and with a highly marked diurnal rhythm during daytime [35] and could be involved in maintaining cell functions during prolonged light exposure. *Pax4* is governed by a set of transcription factors, including members of the orthodenticle family of homeobox genes, such as *Otx2* and *Crx* [75]–[77]. *Crx* is regulated by T3 and therefore disk and opsin renewal [78] could be controlled by the thyroid hormone cascade through activation of *Pax4* and *Crx*. Moreover, *Ccng1* and *Cpt1a* could be playing a role in protection and metabolism in the retina and be controlled by light through the activation of the thyroid hormone cascade [49], [51], [52].

## Materials and Methods

### Ethics Statement

Experiments were supervised and authorized by the SISSA Ethics Committee (Prot.n. 2190 II/7). All rat experiments were carried out according to the Italian and European guidelines for animal care (d.l.116/92; 86/609/C.E.).

### Harvesting rat retinas and culture rat retinas

Dark-adapted Long Evans male adult rats were sacrificed under an infrared light source. The harvested retinas were expelled into 500 µl of TRI Reagent T9424 (Sigma Aldrich) on ice and stored at −80°C. Culture retinas were prepared as described on the protocol used by Reidel et al [31].

### Immunohistochemistry

Immunolabeling was performed by standard protocols for tissue fixation and processing, using as primary antibody anti-Dio2 sc-98716 (Santa Cruz Biotechnology, Inc) and DAPI (Boehringer Mannheim GmbH, Germany) for nuclear staining.

### Western blotting

Retinas, dissected from light or dark-exposed mice, were homogenized in Lysis buffer (50 mM Tris pH 7.5; 150 mM NaCl; 1% Triton X−100; 10 mM MgCl_2_) in ice. The total amount of protein was determined by using BCA protein assay kit (Pierce Biotechnology). The homogenate was diluted in a sample buffer (20μg), run on SDS-PAGE and western blotted using the following antibodies: anti-Dio2 sc-98716 (Santa Cruz Biotechnology, Inc), anti-Dio3 ab82041 (Abcam, Cambridge, UK). β-Actin HRP- conjugated A3854 (Sigma-Aldrich) was used as housekeeping control. Signals were detected analyzing the optical density of the spots.

### Microarray hybridization

Total RNA was purified using the RNeasy mini kit (Qiagen). The RNA quality was checked using a bioanalyzer (Agilent 2100; Agilent Technologies), and the RNA quantity was measured with ND-1000 Nanodrop spectrophotometer. 10 µg of RNA sample was used for microarray analysis on Affymetrix RAT230_2 GeneChip containing 31099 probes, corresponding to 14181 probes with a gene symbol (Affymetrix). Low level analysis was performed using an Robust Multi-array Average (RMA) algorithm (Irizarry et al., 2003) directly on the scanned images. All data is MIAME compliant and that the raw data has been deposited in a MIAME compliant database (E.g. ArrayExpress, GEO), as detailed on the MGED Society website http://www.mged.org/Workgroups/MIAME/miame.html.

### Analysis of microarray data

Data were organized in matrices ‘‘m×n’’ (m, number of genes; n, number of replicas). Five samples were considered: a control at 7 am in dark (Cij; i = 1,...,n; j = 1,...,m), a sample always kept in dark till 10 am (3Dij), a sample with 3 hours of continuous light also at 10 am (3Lij), and two similar samples at 1 pm (6Dij, 6Lij). Data were analyzed by considering log2 changes in gene expression in each replica against the control condition C, that is, log2(3Dij/Cij), log2(3Lij/Cij) and log2(6Dij/Cij), log2(6Lij/Cij). Up-regulated genes for each replica were obtained by selecting all genes showing an up-regulation higher than 60%. Down-regulated genes were obtained considering genes with a decrease of expression larger than 0.7. Intersection between the three replicas was performed and presented in Fig.1. Thus, from the microarray data we obtained an ‘‘m×n’’ ratio-matrix for each condition. Considering the three replicas as independent variables, this matrix was treated as a multivariate variable in three dimensions. We derived the empirical cumulative distribution function with upper and lower bounds of the multivariate variable, using the Kaplan–Meier estimator (Kaplan and Meier, 1958) so to assign a P-value to all the genes and select the most significant ones.

### Real-time PCR on retinas

Long Evans rats were bred and maintained under a 12 hour light/dark cycle (7 AM:7 PM). For changes of lighting environment, two groups of overnight dark-adapted animals were maintained in either a darkened or a lighted cage. A 60 W bulb was used as an adjustable light source. For each time point at least six animals were sacrificed by CO_2_ inhalation, the eyes enucleated, the lenses removed and the retinas collected in TRI Reagent (Sigma Aldrich). Total RNA from retinas was extracted according to the manufacturer's instructions (Sigma Aldrich). After resuspension in DEPC-treated H_2_O, RNA was further purified using an RNeasy column (Qiagen) and quantified using an ND-1000 Nanodrop spectrophotometer (Nanodrop Technologies). Total RNA (500 ng) was treated with DNAse I (Invitrogen) to remove any genomic DNA contamination and converted to cDNA using Superscript II reverse transcriptase (Invitrogen). Twenty microliter PCR reaction mixtures contained cDNA, SYBR green master mix (BioRad), H_2_O, and custom primers designed for each gene of interest. The PCR reactions were performed in an iQ5 thermocycler (BioRad). Each reaction was performed at least in duplicate, and threshold cycles (Ct) were calculated using the second derivative of the reaction. The Ct of each gene was normalized against that of the control reference transcript *Gapdh*. The variation of *Gapdh* in Ct between samples obtained from rats exposed to darkness and to light showed no significant difference. For the experiments shown in Fig. 5 two housekeeping genes were used *Gapdh* and *Hprt*. Normalization was performed using the geometrical mean as described by Vandesompele et al [79]. Both of them showed no difference in Ct. Fold changes were determined using the comparative Ct method (2^−ΔΔCt^ method), using the average of dark control set to one [80]–[82]. RNA controls were performed to ensure that amplification of products did not come from genomic DNA contamination.

### Primers used for Real-Time PCR

Primers shown in Table 1 were used for Real-Time-PCR.

**Table 1:** Primers used for the real time PCR.

Primer name	Sequence
*Gapdh*	F: 5’- CAAGTTCAACGGCACAGTCAAGG -3’R: 5’- ACATACTCAGCACCAGCATCACC -3’
*Hprt*	F: 5’- TTGTTGGATATGCCCTTGACT -3’R: 5’- CCGCTGTCTTTTAGGCTTTG -3’
*Dio2*	F: 5’- TTATGGGGTAGCCTTTGAACG -3’R: 5’- CCAGCCAACTTCGGACTTC -3’
*Dio3*	F: 5’- GCTGTGCTCTGGTTCTGGAC -3’R: 5’- GATGGTGCCGCTCTGGATG -3’
*Opn1mw*	F: 5’- AGGATAGCACCCAGGCAAGCAT -3’R: 5’- GTGCTGGTGAGGTGGTACACCC -3’
*Opn1sw*	F: 5’- GGACTTACGGCTTGTCACCATCCC -3’R: 5’- TGTCATGGGCTTCCTGCACACC -3’
*Sag*	F: 5’- TGCTCAGTGATGTTGCAGCCAGC -3’R: 5’- CCGCACAGAGCTCTTCTTGGGGA -3’
*Gcap1*	F: 5’- GATCGACATCAACGGGGATGGGG -3’R: 5’- GCGGGTCAAGTCCAGGCTTCG -3’
*Ccng1*	F: 5’- GGCGTGCCACTGCAGGATCAT -3’R: 5’- TTCAGTGCTTGGATCTCCAAAGCGA -3’
*Cpt1a*	F: 5’- CGCATGACAGCACTGGCCCAG -3’R: 5’- ACTCACGTAATTTGTGGCCCACCA -3’

F: forward primer, R: reverse primer.

## Supporting Information

Table S1 **Relative abundance of Dio2 and Dio3.** From the data presented in Figure 2, using the ΔCt method and as reference the housekeeping genes, the relative abundance of Dio2/Dio3 were calculated. The time points represent the values of circadian rhythm, that is, at 0, 12, 16 and 20 ZT in dark condition and 4, and 8 ZT under normal ambient illumination.
(DOC)
