# Supplementary material for: A Component of Retinal Light Adaptation Mediated by the Thyroid Hormone Cascade
Source: PLoS One. 2011 Oct 24;6(10):e26334. doi: 10.1371/journal.pone.0026334 (PMC3200322; doi:10.1371/journal.pone.0026334)
Supplement: Table S1 — Relative abundance of Dio2 and Dio3. From the data presented in Figure 2, using the ΔCt method and as reference the housekeeping genes, the relative abundance of Dio2/Dio3 were calculated. The time points represent the values of circadian rhythm, that is, at 0, 12, 16 and 20 ZT in dark condition and 4, and 8 ZT under normal ambient illumination. (DOC) [file pone.0026334.s001.doc]

| **Time** | **Dio2** | **Dio3** | **Dio2/Dio3** |
| --- | --- | --- | --- |
| 0 ZT | 0.001 | 0.003 | 0.395 |
| 4 ZT | 0.010 | 0.001 | 7.552 |
| 8 ZT | 0.010 | 0.001 | 10.324 |
| 12 ZT | 0.003 | 0.004 | 0.767 |
| 16 ZT | 0.003 | 0.007 | 0.373 |
| 20 ZT | 0.003 | 0.004 | 0.703 |
